# Whole-Genome Sequencing-Based Characterisation of Clinically Relevant Mycobacterium abscessus Complex Isolates from India

**DOI:** 10.7759/cureus.111934

**Published:** 2026-07-02

**Authors:** Usha Kattamanchi, Rakesh Kodati, Neelima Angaali, Patil Madhusudhan, Vishnukanth Govindaraj, Gongati Kruparao Paramjyothi, Noyal Joseph Mariya, Kumar Ebineshan

**Affiliations:** 1 Department of Microbiology, Jawaharlal Institute of Postgraduate Medical Education and Research (JIPMER), Pondicherry, IND; 2 Department of Pulmonology, Nizam's Institute of Medical Sciences (NIMS), Hyderabad, IND; 3 Department of Microbiology, Nizam's Institute of Medical Sciences (NIMS), Hyderabad, IND; 4 Department of Pulmonary Medicine, Jawaharlal Institute of Postgraduate Medical Education and Research (JIPMER), Pondicherry, IND; 5 Department of Microbiology, Blue Peter Public Health and Research Centre (BPHRC), Lepra Society, Hyderabad, IND

**Keywords:** antimicrobial resistance, mycobacterium abscessus, non-tuberculous mycobacteria, pulmonary infection, whole genome sequencing

## Abstract

Background: *Mycobacterium abscessus* complex is a rapidly growing non-tuberculous mycobacterium(NTM) associated with difficult-to-treat pulmonary and extrapulmonary infections. Clinical management is complicated by intrinsic antimicrobial resistance, inducible macrolide resistance, and genomic heterogeneity. This study characterised five clinical pulmonary *M. abscessus* isolates from India using whole-genome sequencing (WGS)-based genomic, phylogenetic, and antimicrobial resistance analysis.

Methods: Five clinical isolates recovered from pulmonary specimens were sequenced using the Illumina NovaSeq platform (Illumina, Inc., San Diego, California, United States) with paired-end 151 bp chemistry. Reads were quality filtered using fastp v1.0.1. Reference-guided alignment and consensus generation were performed using Burrows-Wheeler Aligner (BWA)-Maximal Exact Match (MEM) and SAMtools-based workflows. Genome annotation was conducted using Prokka v1.14.6, functional annotation using eggNOG-mapper, variant analysis using Genome Analysis Toolkit (GATK) HaplotypeCaller and SnpEff, and antimicrobial resistance gene detection using the Comprehensive Antibiotic Resistance Database (CARD). Phenotypic antimicrobial susceptibility findings and GenoType NTM-drug resistance (DR) Line Probe Assay Version 1.0 results (Bruker Corporation, Billerica, Massachusetts, United States) were used to support the interpretation of macrolide resistance-associated findings.

Results: The isolates generated 0.92-1.24 Gb of high-quality sequencing data, with Q30 values exceeding 95%. Genome coverage at ≥30× depth exceeded 91%, and average sequencing depth ranged from 44.16× to 149.59×. Annotation identified 4,457-4,751 coding sequences, 45-48 tRNA genes, three rRNA genes, and one tmRNA gene per isolate. Reference alignment ranged from 24.86% to 64.22%, supporting isolate-level genomic heterogeneity. MAB β-lactamase (*blaMab*) gene was detected in all isolates, while *erm(41)* was detected only in LIN.

Conclusion: WGS-enabled isolate-level characterisation of pulmonary* M. abscessus* isolates from India demonstrated conserved genomic features with measurable heterogeneity. *blaMab* supports intrinsic β-lactam resistance, while *erm(41)* in LIN was consistent with an inducible macrolide resistance pattern supported by available phenotypic antimicrobial susceptibility testing.

## Introduction

In recent years, non-tuberculous mycobacteria (NTM) have become clinically important opportunistic pathogens associated with pulmonary, extrapulmonary, and disseminated infections, particularly in susceptible hosts. Diagnosis and management remain challenging because NTM persist in environmental reservoirs, show variable antimicrobial susceptibility, and differ in clinical relevance at the species and subspecies levels. Genospecies identification is important in clinically relevant mycobacterial disease [1]. The *Mycobacterium abscessus* complex is particularly important among NTM because it is associated with difficult-to-treat pulmonary infection, intrinsic multidrug resistance, prolonged treatment requirements, and poor therapeutic outcomes. Human-transmissible and multidrug-resistant lineages have also been reported, emphasizing their relevance to clinical microbiology, infection control, and genomic surveillance [2].

Whole-genome sequencing (WGS) provides high-resolution characterization of clinically relevant mycobacteria by enabling assessment of strain diversity, antimicrobial resistance determinants, genomic variation, and phylogenetic relationships [3,4]. When interpreted through validated genomic analysis frameworks, WGS can support integrated assessment of genome structure, functional gene content, resistance-associated determinants, and isolate relatedness [5,6]. In *M. abscessus*, genome-based analyses are especially relevant because intrinsic, inducible, and acquired resistance mechanisms can affect antimicrobial interpretation and therapeutic planning [7].

Environmental persistence contributes substantially to the clinical burden of NTM. These organisms are widely distributed in water sources and may cause community-acquired or healthcare-associated infection [8]. Repeated exposure, colonization, and infection are more likely in individuals with chronic lung disease, structural airway abnormalities, previous tuberculosis, bronchiectasis, cystic fibrosis, or immune deficiency. Accurate species identification and genomic characterization can support diagnosis, strain-relatedness assessment, transmission evaluation, and healthcare-associated risk assessment. Genome annotation and comparative genomic analysis can identify coding sequences, RNA genes, functional pathways, and genomic features related to metabolism, cell wall biology, stress response, efflux, persistence, and antimicrobial resistance [9]. These features are relevant because the rise of *M. abscessus* as an important NTM pathogen has been linked to environmental adaptation, intracellular survival, biofilm formation, disinfectant tolerance, antibiotic resistance, and chronic pulmonary disease [10].

Genome-based bacterial taxonomy continues to evolve, with implications for the *M. abscessus* complex because subspecies-level differences may affect macrolide susceptibility and inducible resistance [11]. Culture-based mycobacterial diagnosis remains fundamental, and genomic findings should be interpreted alongside appropriate culture methods, laboratory identification, phenotypic susceptibility testing, and clinical context [12]. Although the clinical significance of NTM pulmonary disease is increasing in India, genomic information on clinical* M. abscessus* isolates remains limited. This study aimed to perform WGS-based characterization of five clinical* M. abscessus* complex isolates recovered from pulmonary specimens in India, with emphasis on sequencing quality, genome annotation, functional profiling, antimicrobial resistance determinants, variant analysis, and phylogenetic relationships.

Objectives of the study

This study aimed to perform a descriptive WGS-based characterization of five clinical *M. abscessus* complex isolates recovered from pulmonary specimens in India. The exploratory genomic objectives were to assess sequencing quality, genome annotation features, functional genomic profiles, variant patterns, and phylogenetic relationships among the clinical isolates and reference strains. The clinically oriented objective was to identify antimicrobial resistance-associated genes and interpret these findings alongside available phenotypic antimicrobial susceptibility results. Given the limited sample size and absence of linked patient-level clinical outcome data, the objectives were framed as isolate-level genomic characterization rather than population-level epidemiological or clinical outcome inference.

## Materials and methods

Study design and clinical isolates

This was a laboratory-based descriptive WGS study for the characterisation of *M. abscessus* complex clinical isolates obtained from pulmonary specimens in India. Five clinical isolates were included and identified as NAG, YAD, LIN, SUS, and YAS. Genomic analysis was performed after the isolates were identified as part of the *M. abscessus* complex using routine clinical microbiology workflows before the onset of genomic analysis. The study was designed to generate isolate-level genomic data rather than population-level epidemiological estimates. The study provided a focus on genomic characterisation of the isolates at the isolate level, such as assessing sequencing quality, reference genome analysis, genome annotation, functional profiling, detecting variants, screening for antimicrobial resistance genes, and phylogenetic analysis of genomes. The present analysis did not involve patient-level clinical variables, treatment history, radiological findings, or clinical outcomes. In genome-based taxonomic classification, *Mycobacteroides abscessus* is the recognised nomenclature, but the former *Mycobacterium abscessus* complex was retained in this study due to the continued use of this term in clinical and medical literature [13].

Ethical considerations

The study protocol was approved by multiple Institutional Ethics Committees (IECs) in India. Ethical clearance was obtained from the LEPRA Society-IEC, Hyderabad, India (approval ID: 10/LEPRA/IEC/2024-2025). Additional approvals were obtained from the IEC of Jawaharlal Institute of Postgraduate Medical Education and Research (JIPMER), Puducherry, India (project number: JIP/IEC-OS/222/2023), and the IEC of Nizam’s Institute of Medical Sciences (NIMS), Hyderabad, India (review letter number: EC/NIMS/3717/2025).

DNA sequencing

Five clinical isolates were subjected to WGS with the Illumina NovaSeq platform (Illumina, Inc., San Diego, California, United States) using paired-end 151 bp chemistry, and genomic DNA was extracted from the isolates. Sequencing was performed using short-read paired-end sequencing to support reference-guided genome alignment, genome coverage assessment, annotation, variant detection, antimicrobial resistance gene screening, and comparative genomic analysis. The sequencing strategy resulted in short read sequences that are appropriate for reference genome-based analysis, short-read coverage, genome annotation, variant detection, antimicrobial resistance gene screening, and comparative genome analysis. Next-generation sequencing was employed as it allows for high-resolution sequencing of bacterial genomes, genomic diversity, and clinically important genetic variants [6,14].

Quality control and read processing

The raw FASTQ files were evaluated for quality of base, guanine-cytosine (GC) content distribution, level of sequence duplication, adapter contamination, and read quality. The adapter trimming and quality filtering were done with fastp v1.0.1. Any reads that failed quality control and adapter-contaminated reads were removed before downstream analysis. Only high-quality filtered reads were used for genome alignment and genomic characterisation. The quality of sequencing was summarised by total read count, total data output, GC content, and Q30 percentage. Sequencing adequacy was interpreted using total read count, total data output, GC content, Q30 percentage, reference alignment percentage, genome coverage at ≥30× depth, and average sequencing depth. These metrics were used to determine whether the sequencing data were suitable for descriptive isolate-level genomic analysis.

Reference-guided genome alignment and consensus generation

The reads were quality filtered, and alignable reads were mapped to the reference genome strain of *M. abscessus* FLAC049 using Burrows-Wheeler Aligner (BWA)-Maximal Exact Match (MEM). Burrows-Wheeler transform-based mapping techniques are popular in the field of high-throughput genome analysis for efficient short-read sequencing data alignment [11,12]. Sorting and indexing of mapped reads was performed as part of alignment processing, which was done with SAMtools workflows. SAMtools and BCFtools are well-known tools that process alignment and variant data in genome-sequencing studies [5].

Each isolate was sequenced, and consensus genome sequences were generated from the reference-aligned reads. The genome coverage and sequencing depth were determined by BEDTools (https://bedtools.readthedocs.io/en/stable/) and custom Perl scripts. All isolates were also sequenced to assess sequencing data for comparative genomic interpretation by recording reference alignment percentage, genome coverage at ≥30× depth, and average sequencing depth. Reference-guided outputs were interpreted descriptively, with attention to the possibility that reference divergence, accessory genome content, or strain-level variation could affect alignment percentage and genome representation.

Genome annotation

Prokka v1.14.6 (https://gensoft.pasteur.fr/docs/prokka/1.14.6/) was used to predict coding sequences, transfer RNA (tRNA) genes, ribosomal RNA (rRNA) genes, and transfer-messenger RNA (tmRNA) genes by annotation of the genome. Prodigal (https://github.com/hyattpd/prodigal) was also used to assist with gene prediction. Predicted gene content was compared across isolates by using annotation outputs to aid downstream functional analysis. High-quality genome annotation of bacterial genes is needed for understanding the coding potential, distribution of functional genes, and isolate-specific genomic features [9]. Annotation results were interpreted as predicted genomic features and were not considered direct evidence of biological function without phenotypic or transcriptomic validation.

Functional annotation

The functional annotation was done by EggNOG-mapper [15]. Predicted genes were categorised with the Cluster of Orthologous Groups [16], the Kyoto Encyclopedia of Genes and Genomes database [17], and the Gene Ontology database [18]. Genes belonging to functional categories were evaluated to identify genes annotated in categories related to bacterial persistence and host-associated survival mechanisms, including metabolism, environmental adaptation, stress response, cell wall biosynthesis, and other biological processes. These functional analyses were applied to the interpretation of the genome descriptively. None of the functional phenotypes was derived from the annotation results alone. Therefore, functional annotation was used only to describe predicted gene categories and not to infer confirmed pathogenicity, persistence, environmental adaptation, or virulence phenotypes.

Variant calling and variant annotation

The reference-aligned Binary Alignment Map (BAM) files were used to call variants with Genome Analysis Toolkit (GATK) HaplotypeCaller [19]. SnpEff (https://pcingola.github.io/SnpEff/) was used to annotate variants. Single-nucleotide polymorphisms (SNPs) and insertion/deletion events (INDELs) were detected in coding and non-coding regions. A variant call format (VCF) file, with annotations, was created for each isolate to determine the genomic variation between them and the chosen reference genome. Only genomic characterisation was considered a method for variant interpretation. Variants were not assumed to represent clinically relevant markers of antimicrobial resistance unless they had been associated with known antimicrobial resistance annotations or previously defined resistance-associated genes. This conservative approach was adopted as genomic testing for the detection of variants is not a substitute for phenotypic antimicrobial susceptibility testing. Variant findings were interpreted as descriptive isolate-level genomic variation rather than clinically actionable resistance markers unless supported by recognised resistance annotations or phenotypic susceptibility evidence.

Antimicrobial resistance gene analysis

The Comprehensive Antibiotic Resistance Database (CARD) was used to identify antimicrobial resistance genes [20]. The detection of antimicrobial resistance (AMR)-associated genes was analysed based on the predicted resistance mechanism, the associated antimicrobial class, and the sequence-level similarity. Focus was placed on genes associated with β-lactam resistance and inducible macrolide resistance, as these are clinically significant in the treatment of *M. abscessus* complex infections [7]. Resistance mechanisms were characterised with the aid of curated annotations from the database. Putative resistance determinants were considered to be genomic AMR results. Phenotypic antimicrobial susceptibility testing was available and was used to support the interpretation of the genomic antimicrobial resistance findings, particularly the inducible macrolide resistance pattern observed in the LIN isolate. In addition, the GenoType NTM-DR Line Probe Assay Version 1.0 (Bruker Corporation, Billerica, Massachusetts, United States) was used for the detection of resistance-associated markers, including *erm(41)*-associated macrolide resistance determinants. Genomic AMR findings were interpreted alongside available phenotypic susceptibility evidence and were not used alone as definitive predictors of clinical treatment response.

Comparative genome and phylogenetic analysis

Comparative genome analysis was performed to evaluate genomic relatedness among the five clinical isolates and selected reference strains. Whole-genome-based comparisons were used to assess genome conservation, isolate-level divergence, and clustering patterns. Phylogenetic findings were interpreted in relation to reference alignment percentage, genome coverage, and observed genomic variation. Because the *M. abscessus* complex includes clinically relevant subspecies with potential differences in antimicrobial susceptibility, especially macrolide resistance, phylogenetic interpretation was treated as exploratory unless supported by dedicated subspecies-level classification. Genome-enabled subspecies identification is increasingly important for interpreting the clinical and microbiological diversity of the *M. abscessus* complex. The phylogenetic analysis included selected *M. abscessus *complex reference strains, including T927, FLAC, FDAA, and GD38, for comparison with the five clinical isolates. *Mycobacterium tuberculosis* was included as an outgroup/comparator to provide evolutionary context, facilitate interpretation of tree topology, and distinguish clustering within the *M. abscessus* complex from broader mycobacterial relatedness. Subspecies-level assignment was not interpreted definitively because a validated, dedicated subspecies typing workflow was not included in the present analysis.

Data management and analytical approach

Isolate-level quality measures, genome annotation results, functional annotation files, VCF files, AMR profile files, and comparative genomic output files were collected. The results were summarised descriptively since the study involved five isolates and was designed as a focused genomic characterisation study and not a population-level epidemiological study. No statistical tests of inference were performed. The analysis was therefore limited to descriptive isolate-level genomic characterization, with clinical interpretation restricted to available phenotypic antimicrobial susceptibility findings and without inference regarding treatment outcomes, transmission dynamics, or population-level epidemiology.

## Results

Sequencing quality metrics

All five clinical *M. abscessus* complex isolates generated sequencing data suitable for descriptive whole-genome analysis. Total sequencing output ranged from 0.92 Gb to 1.24 Gb per isolate, with total reads ranging from 6,077,714 in YAD to 8,231,346 in NAG. The Q30 value exceeded 95% in all isolates, supporting reliable base-call accuracy for descriptive genomic interpretation. The percentages for reference alignment of isolates ranged from 24.86% to 64.22% for SUS and NAG, respectively. NAG and YAD demonstrated the highest reference alignment percentages (64.22% and 64.20%, respectively), indicating closer similarity to the selected reference genome. The lowest reference alignment percentage was reported for SUS, which maintained genome coverage of at least 30X above 91%, which may indicate divergence in the reference genome, accessory genome, or strain-level genomic differences. The average sequencing depth of the samples ranged from 44.16X in SUS to 149.59X in NAG (Table 1).

**Table 1:** Sequencing and assembly statistics GC: guanine-cytosine

Isolate	Total Reads	Total Data (Gb)	GC (%)	Q30 (%)	Reference Alignment (%)	Coverage ≥30× (%)	Average Depth
NAG	8,231,346	1.24	56.77	95.30	64.22	93.39	149.59×
YAD	6,077,714	0.92	56.74	95.10	64.20	93.38	110.41×
LIN	6,911,534	1.04	55.08	95.85	44.70	94.77	92.77×
SUS	6,350,686	0.96	60.42	95.64	24.86	91.80	44.16×
YAS	6,313,472	0.95	52.39	95.74	49.52	92.43	86.89×

Genome annotation

The five isolates contained 4,457-4,751 coding sequences when annotated to the genome. YAD had the largest number of predicted coding sequences (4,751) and total number of genes (4,803), followed closely by NAG (4,750 CDS and 4,802 total genes). LIN contains the minimum number of coding sequences (CDS) and total genes (4,457 CDS and 4,506 total genes). All isolates contained three rRNA genes and one tmRNA gene. The number of tRNA genes varied from 45 to 48, with NAG and YAD containing the highest number of tRNA genes. The trRNA gene number varied from 45 to 48, NAG and YAD containing the maximum number of trRNAs (Table 2).

**Table 2:** Genome annotation statistics CDS: coding sequences

Isolate	CDS	Total Genes	rRNA	tRNA	tmRNA
NAG	4,750	4,802	3	48	1
YAD	4,751	4,803	3	48	1
LIN	4,457	4,506	3	45	1
SUS	4,712	4,763	3	47	1
YAS	4,629	4,678	3	45	1

Functional annotation

The prediction of genes was functionally annotated and showed that the five isolates represent a wide range of core bacterial metabolic and adaptive processes. Among the major functional categories, cellular metabolism, amino acid transport and metabolism, lipid metabolism, energy production and conversion, environmental adaptation, stress response, and cell wall biosynthesis were the predominant categories. The categories are important for bacterial viability, bacterial use of the nutrients, persistence, and adaptation to environmental or host-associated factors. Genes associated with biological processes, molecular functions, and cellular components were identified by using the gene ontology-based classification. Functional annotation is interpreted descriptively, as there were no quantitative counts of isolates at the category level. The overall functional profile identified annotated gene categories related to metabolism, cell envelope biology, stress response, and other bacterial processes; however, these findings were interpreted descriptively and were not considered direct evidence of functional phenotype without phenotypic or transcriptomic validation (Table 3).

**Table 3:** Major functional categories identified across the five isolates

Functional Category	Representative Biological Relevance	Isolates Detected
Cellular metabolism	Core growth, viability, and biosynthetic activity	LIN, NAG, SUS, YAD, YAS
Amino acid transport and metabolism	Nutrient acquisition and metabolic adaptation	LIN, NAG, SUS, YAD, YAS
Lipid metabolism	Cell envelope structure and mycobacterial membrane biology	LIN, NAG, SUS, YAD, YAS
Energy production and conversion	Respiratory activity and energy homeostasis	LIN, NAG, SUS, YAD, YAS
Environmental adaptation	Survival under variable environmental conditions	LIN, NAG, SUS, YAD, YAS
Stress response	Tolerance to host-associated and environmental stressors	LIN, NAG, SUS, YAD, YAS
Cell wall biosynthesis	Maintenance of mycobacterial cell wall architecture	LIN, NAG, SUS, YAD, YAS

Variant analysis

Five isolates showed variation in their genome, with both SNPs and insertion/deletion events found within both coding and non-coding regions, through variant analysis. All isolates were also annotated, and the VCF files were used to assess the genomic differences between individual isolates and the chosen reference genome. This detected variation enabled the detection of measurable genomic heterogeneity between the clinical isolates analysed.

Descriptive genomic characterisation was the only level of variant interpretation achieved. Variants were not considered clinically actionable unless there was known resistance annotation or known resistance-associated genes. The annotated VCF outputs included isolate-level variant counts and predicted impact categories. The alignment percentage of the reference was very low in SUS, indicating the presence of divergent regions in the genome or regions that were not covered by the reference-guided workflow, which is why the genome coverage at ≥30× depth was still present.

AMR gene analysis

All five isolates were screened for AMR genes and were found to contain the class A β-lactamase gene (MAB). This discovery is in agreement with the existence of a conserved genomic determinant linked to intrinsic β-lactam resistance in the analysed isolates. Associated β-lactam antibiotics were ceftazidime, cefuroxime, amoxicillin, cefalotin, and ticarcillin. Only the LIN isolate was positive for *erm(41)*, an inducible macrolide resistance gene. Phenotypic antimicrobial susceptibility testing was available and demonstrated an *erm(41)*-associated resistance pattern suggestive of inducible macrolide resistance in the LIN isolate. The finding indicates that LIN might have genomic potential for inducible macrolide resistance via target modification. The macrolide antibiotic drugs that were associated were clarithromycin, azithromycin, and erythromycin. Using the GenoType NTM-DR Line Probe Assay Version 1.0, only the LIN isolate demonstrated the *erm(41)* T28 genotype, while the remaining isolates showed no resistance-associated mutations. A homolog of the VanY type was found in all isolates. This homolog was linked to glycopeptide resistance annotations (Table 4).

**Table 4:** Comparative AMR gene profile AMR: antimicrobial resistance

Isolate	Detected AMR Genes	Predicted Resistance Mechanism	Associated Antibiotic Classes/Agents	Interpretation
LIN	*erm(41)*, *blaMab*, vanY-like	Target modification; antibiotic inactivation	Macrolides, β-lactams, vancomycin	Presence of *erm(41)* suggests possible inducible macrolide resistance; *blaMab *supports intrinsic β-lactam resistance.
NAG	*blaMab*, vanY-like	Antibiotic inactivation	β-lactams, vancomycin	*blaMab* supports intrinsic β-lactam resistance; vanY-like findings have limited clinical interpretability
SUS	*blaMab*, vanY-like	Antibiotic inactivation	β-lactams, vancomycin	AMR profile similar to NAG; vanY-like finding likely represents a distant homolog
YAD	*blaMab*, vanY-like	Antibiotic inactivation	β-lactams, vancomycin	Conserved *blaMab*-associated β-lactam resistance determinant detected
YAS	*blaMab*, vanY-like	Antibiotic inactivation	β-lactams, vancomycin	AMR profile similar to NAG and SUS

The reported low sequence identity of ~26-27% suggests that there is little clinical interpretability. These findings are more likely to represent a distant homolog of vanY rather than true evidence of functional vancomycin resistance (Table 5).

**Table 5:** Comparative summary of key AMR genes AMR: antimicrobial resistance

Gene	LIN	NAG	SUS	YAD	YAS
erm(41)	Present	Absent	Absent	Absent	Absent
blaMab	Present	Present	Present	Present	Present
vanY-like	Present	Present	Present	Present	Present

Comparative genome mapping and phylogenetic analysis

**Figure 1:**
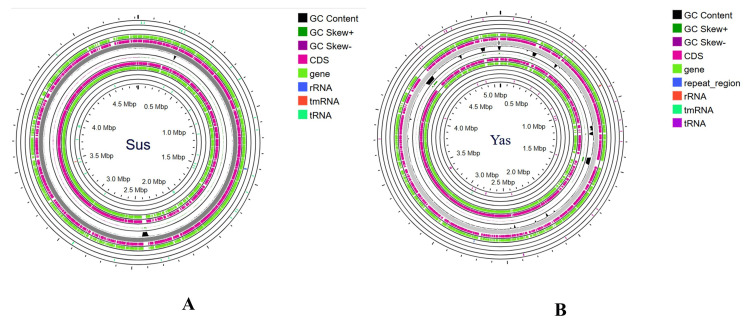
Representative circular genome maps of (A) SUS and (B) YAS showing genome organisation and genomic features GC: guanine-cytosine

Whole genome analysis showed a high degree of conservation of genome organisation with differences at the level of the isolate in the five clinical isolates of the *M. abscessus *complex. Genome maps of circular structure were generated, and coding sequences, GC content, GC skew, rRNA genes, tRNA genes, tmRNA genes, and repeat regions were shown to be distributed across the genomes. The genome architecture of all the isolates was found to be similar, and certain differences in the distribution of repeats and GC skew indicated genomic diversity intraspecifically within the complex. Circular genome maps showed key genomic features in SUS (Figure 1A) and YAS (Figure 1B).

**Figure 2:**
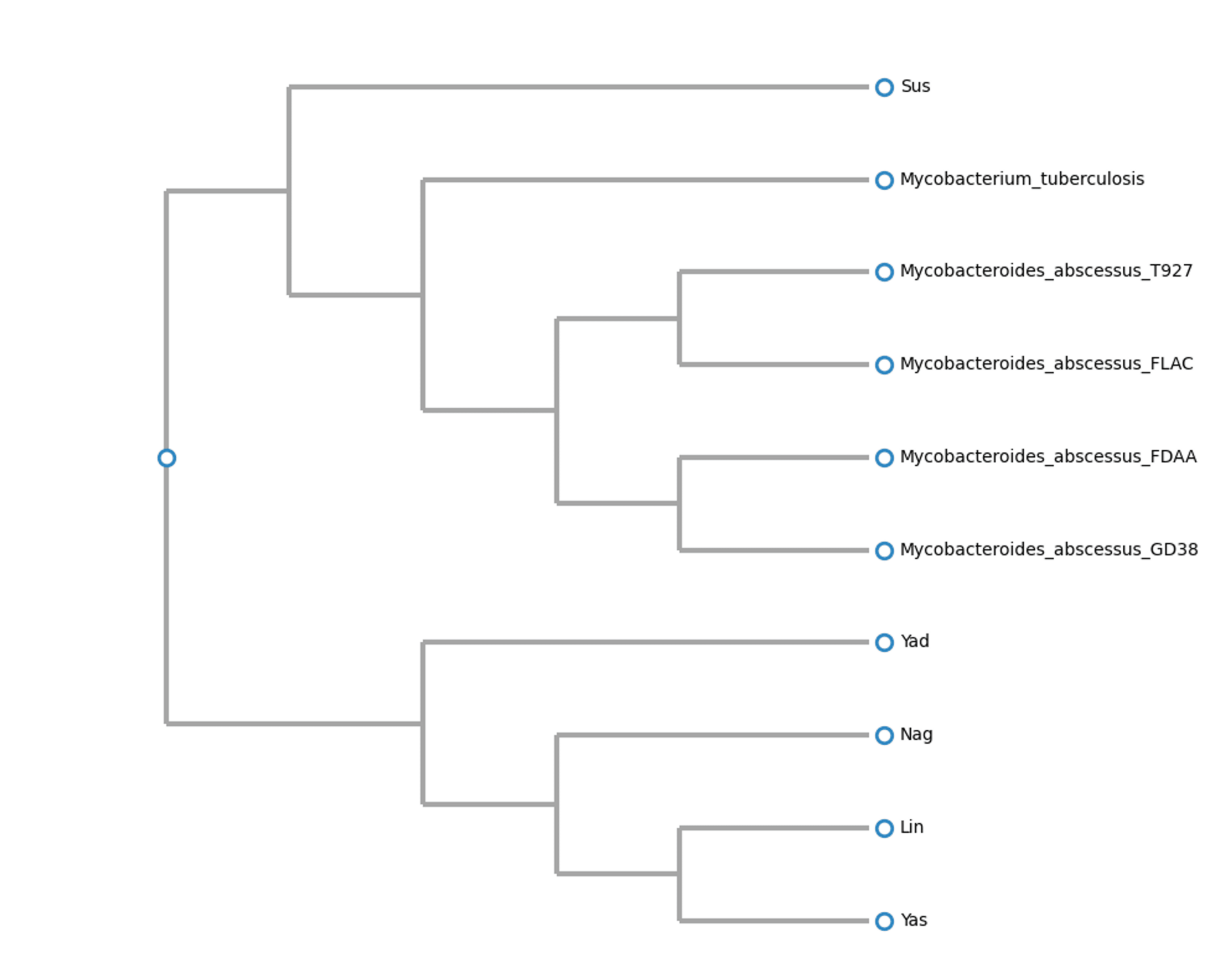
Phylogenetic tree of clinical Mycobacterium abscessus complex isolates and reference strains

All five clinical isolates were found to be within the *M. abscessus* complex using whole-genome-based phylogenetic analysis. The subspecies level was not considered to be definitive when not part of a subspecies assignment workflow. SUS was grouped with the reference strains T927, FLAC, FDAA, and GD38, suggesting a relationship within the group of reference strains. YAD, NAG, LIN, and YAS came together in a group. In this cluster, the closest relation was between LIN and YAS, while NAG grouped close to them, and YAD was in a relatively isolated branch. Phylogenetic analysis revealed that the SUS group was near the reference strains, and LIN was close to the YAS group. *M. tuberculosis* was included as an outgroup/comparator to provide evolutionary context and facilitate interpretation of the tree topology. As a distinct but clinically relevant mycobacterial species, it helped distinguish clustering within the *M. abscessus* complex from broader mycobacterial relatedness (Figure 2).

The five clinical isolates were found to have conserved genome-level features, but also measurable isolate-level diversity, overall suggesting that the five isolates have common features at the genome level, and yet are distinct in terms of measurable diversity. The genomic heterogeneity of Indian isolates of the pulmonary *M. abscessus* complex is supported by variation in reference alignment, clustering patterns, and gene content of the AMR. Because of the limited number of isolates studied (five), in the absence of phenotypic antimicrobial susceptibility testing and clinical outcome data at the patient level, these results should be interpreted as descriptive, isolate-level results, and not as population-level estimates of epidemiology.

## Discussion

In this study, WGS was used to characterise five clinical *M. abscessus* complex isolates obtained from pulmonary specimens from India. Clinical relevance of NTM is investigated at the isolate level with the use of WGS sequencing, genome annotation outputs, profiles of AMR genes, and phylogenetic analysis, all of which have been identified as useful tools. The study of bacterial pathogen genomics has grown to be a key factor in research on infectious disease due to its high resolution of pathogen diversity, resistance mechanisms, genetic relatedness, and evolution [14]. In this study, WGS allowed a comparison of genome quality, the prediction of gene content and functional profiles, resistance-associated determinants, and phylogenetic clustering.

Results are to be read in the context of clinical mycobacteriology diagnosis. Culture and identification still play a key role in patient care; however, the mycobacterial diagnosis will require the use of suitable culture media, culture conditions, and careful interpretation of results, due to variations in growth characteristics and clinical importance among the species [21]. All five isolates generated sequencing data with >95% Q30 scores and genome coverage >91% at ≥30× depth. These metrics support suitability for descriptive genomic analysis. The difference in reference alignment percentage between the isolates, especially the lower alignment percentage seen in SUS, could be due to genomic differences from the selected reference strain, accessory genome changes, or mapping bias due to the alignment of the reference.

Although subspecies-level classification is not clinically relevant for all species, for the *M. abscessus* complex, subspecies-level classification is relevant because the genomic structure and antimicrobial susceptibility, in particular the macrolide susceptibility, can differ between them. The use of genome-based approaches has facilitated the classification of the three subspecies of *M. abscessus* and could aid in the interpretation of clinical isolates in a more precise manner [22]. All the isolates in this study were identified as belonging to the M. abscessus complex using phylogenetic analysis. The SUS strains were grouped in proximity to the reference strains, while the cluster of YAS, LIN, and NAG strains was distinct from the other strains, with a close association between LIN and YAS. This pattern reflects the level of genomic heterogeneity in the isolates, but the subspecies assignment should not be taken as definitive, as a dedicated subspecies identification workflow was not performed.

Genes that were associated with metabolism, lipid metabolism, energy production, cell wall biosynthesis, stress response, and environmental adaptation were found during functional annotation. The categories are biologically relevant because the pathogenicity of *M. abscessus* is dependent on its ability to survive in the environment, adapt to the host, survive after being taken up into the host cell, form biofilms, and exhibit a lipid-rich cell envelope [23]. Their distribution across isolates suggests the presence of shared annotated genomic features; however, functional relevance to environmental adaptation, persistence, or pathogenicity cannot be inferred without phenotypic or transcriptomic validation. Phenotypic antimicrobial susceptibility testing provided culture-based evidence supporting the genomic resistance findings, including an inducible macrolide resistance pattern in the LIN isolate.

All the isolates were positive for the presence of the MAB β-lactamase gene (*blaMab*) by AMR analysis, which suggests that the genetic basis of intrinsic β-lactam resistance is conserved. The presence of *erm(41)* was only detected in LIN, as this is the major factor that contributes to the complexity of *M. abscessus* disease for macrolide therapy [24]. The detection of *erm(41)* in the LIN isolate was consistent with phenotypic antimicrobial susceptibility testing, which demonstrated an inducible macrolide resistance pattern. This finding supports concordance between the genomic result and the available culture-based susceptibility evidence for LIN, while the remaining isolates did not show *erm(41)*-associated resistance findings.

The overall significance of these findings is similar to the known environmental properties of *M. abscessus*, which is a persistent, antimicrobial-tolerant, and chronic pulmonary pathogen [25]. Its virulence involves a combination of mechanisms associated with the cell envelope, its ability to survive in cells, biofilm formation, modulation of immunity, and interactions between the host and the pathogen, which may play a role in treatment failure and chronic infection [26]. Caution should be exercised in the interpretation of genome annotation, as the accuracy of the genome depends on the quality of the genome, the extent of the database, and the prediction algorithms used [27]. A total of 4,457 to 4,751 coding sequences were identified in this study by annotation as having conserved features of rRNA, tRNA, and tmRNA. Comparative genome analysis revealed a high level of genome organisation conservation with only small differences in the patterns of repeat sequences and in the GC skew, as found at the strain level in comparative bacterial genomics [28].

The findings have implications for clinical microbiology and genomic surveillance. Laboratory guidance for mycobacteria includes the importance of accurate identification and careful interpretation of culture results, and combining microbiological results with clinical context [29]. These methods can be complemented by WGS, which can provide higher-resolution strain comparisons, detection of resistance genes, and surveillance. The study also indicates that while mutations in common markers are important, mutations in other genes, such as *MAB_2299c*, could lead to cross-resistance to clofazimine and bedaquiline in *M. abscessus* [30]. The main drawbacks are the limited number of isolates, lack of patient-level clinical metadata and clinical outcome data, limited phenotypic susceptibility interpretation, and lack of definitive subspecies assignment. Future large-scale, multi-centre studies including WGS, phenotypic susceptibility testing, subspecies classification, transcriptomics, and clinical outcomes in infections with *M. abscessus* complex will help to resolve the epidemiological and clinical relevance of *M. abscessus* complex genomic diversity.

Strengths, limitations, and future directions

This study has several strengths. It addresses a clinically relevant and underexplored area by applying WGS to characterize clinical *M. abscessus *complex isolates from pulmonary specimens in India. The use of WGS enabled isolate-level assessment of sequencing quality, genomic diversity, annotation profiles, antimicrobial resistance-associated determinants, and phylogenetic relationships. The inclusion of antimicrobial resistance gene analysis and available phenotypic antimicrobial susceptibility findings further strengthened the interpretation of resistance-associated genomic results.

Some limitations to this study should be taken into account when interpreting the results. Only five clinical *M. abscessus* complex isolates were analysed, thus limiting the generalizability of the findings and the ability to make population-level epidemiological inferences. Phenotypic antimicrobial susceptibility testing was performed; however, the phenotypic findings were limited to resistance interpretation and could not be correlated with treatment response because linked patient-level clinical outcome data were unavailable. Therefore, genomic resistance-associated findings should be interpreted cautiously and should not be considered independently predictive of clinical response. Subspecies-level classification was not performed because a validated dedicated subspecies typing workflow was not included in the present analysis, and therefore, subspecies assignment was not interpreted definitively. The comparator/reference strain selection and inclusion of *M. tuberculosis* as an outgroup were used for exploratory phylogenetic context rather than definitive transmission or subspecies-level inference.

More comprehensive isolate collections from various parts of India should be included in future work to more precisely define the genomic diversity, transmission dynamics, and resistance evolution of the *M. abscessus* complex. Combining WGS with expanded phenotypic susceptibility testing, validated subspecies typing, transcriptomic analysis, and comprehensive clinical data would enhance the interpretation of the mechanisms of resistance, pathogenicity, and clinical relevance. Genomic persistence, recurrence, reinfection, and changes during therapy could be better understood using longitudinal sampling pre-, during, and post-therapy from patients. Integrated studies would aid in the enhanced genomic surveillance, laboratory interpretation, and therapeutic decisions of clinically relevant NTM infections.

## Conclusions

This study highlights the potential of WGS to characterise clinical *M. abscessus* complex isolates from pulmonary specimens in India. Within the limits of a descriptive five-isolate study, their sequencing data were suitable for descriptive whole-genome analysis, and the genomes of all five isolates had broadly similar genome organisation, with differences in the reference alignment, annotation profiles, antimicrobial resistance genes present, and clustering of sequences by their respective isolates suggesting measurable genome-level variation at the isolate level. Functional annotation revealed genes related to metabolism, stress response, environmental adaptation, lipid metabolism, and cell wall biosynthesis, although their biological role requires further phenotypic or transcriptomic validation. Intrinsic β-lactam resistance was detected in all isolates by the presence of the *blaMab *gene, which is known to be a conserved genomic determinant of intrinsic β-lactam resistance, and the presence of *erm(41)* was detected only in LIN, consistent with an inducible macrolide resistance pattern supported by phenotypic antimicrobial susceptibility testing.

These resistance findings should be interpreted as genomic and phenotypic observations at the isolate level rather than definitive predictors of clinical treatment response. These findings support the potential utility of WGS as a complementary tool to clinical mycobacteriology for isolate characterization, identification of resistance-associated genes, and genomic surveillance. The phylogenetic findings should be interpreted cautiously because definitive subspecies assignment and transmission inference were not performed. However, larger multicentric studies incorporating transcriptomic analysis, validated subspecies assignment, clinical data, and phenotypic susceptibility testing are required to establish the epidemiological, diagnostic, and therapeutic significance of the genomic diversity of the *M. abscessus* complex in India.
